# Delirium on presentation with a hip fracture is associated with adverse outcomes: a multicentre observational study of 18,040 patients using national clinical registry data

**DOI:** 10.1302/0301-620X.107B4.BJJ-2024-1164.R1

**Published:** 2025-04-01

**Authors:** Rose S. Penfold, Luke Farrow, Andrew J. Hall, Nick D. Clement, Kirsty Ward, Lorraine Donaldson, Antony Johansen, Andrew D. Duckworth, Atul Anand, Daniel E. Hall, Bruce Guthrie, Alasdair M. J. MacLullich

**Affiliations:** 1 Edinburgh Delirium Research Group, Ageing and Health, Centre for Population Health Sciences, Usher Institute, University of Edinburgh, Edinburgh, UK; 2 Advanced Care Research Centre, Usher Institute, University of Edinburgh, Edinburgh, UK; 3 Institute of Applied Health Sciences, University of Aberdeen, Aberdeen, UK; 4 School of Medicine, University of St Andrews, St Andrews, UK; 5 Edinburgh Orthopaedics, NHS Lothian, Edinburgh, UK; 6 Scottish Hip Fracture Audit, Public Health Scotland, Edinburgh, UK; 7 University Hospital of Wales and School of Medicine, Cardiff University, Cardiff, UK; 8 Usher Institute, University of Edinburgh, Edinburgh, UK; 9 Centre for Cardiovascular Sciences, University of Edinburgh, Edinburgh, UK; 10 Wolff Center at UPMC, University of Pittsburgh Medical Center, Pittsburgh, Pennsylvania, USA; 11 Center for Health Equity Research and Promotion, VA Pittsburgh Healthcare System, Pittsburgh, Pennsylvania, USA; 12 GRECC, VA Pittsburgh Healthcare System, Pittsburgh, Pennsylvania, USA; 13 Department of Surgery, University of Pittsburgh, Pittsburgh, Pennsylvania, USA

## Abstract

**Aims:**

Delirium is common in hip fracture patients, but large-scale routine data studies examining the prevalence and associations of delirium at the time of initial presentation with a hip fracture are rare. This study aimed to describe the prevalence and outcomes of delirium on initial presentation with a hip fracture in a large national population sample.

**Methods:**

This study analysed routinely collected national clinical registry data for all people in Scotland aged 50 years and over presenting with a hip fracture between July 2019-December 2021. Delirium was assessed prospectively by clinicians as part of routine care using the 4 ‘A’s Test (4AT; www.the4AT.com), a validated two-minute tool. Associations of 4AT score with mortality and return home within 30 days were analysed using logistic regression models, adjusted for confounders.

**Results:**

Of 18,040 patients (mean age 80 years; 70% women), 16,476 (91%) had a 4AT assessment on presentation and of these, 3,386 (21%) had a score ≥4, suggestive of delirium. Patients with delirium were older, more likely residing in care homes and had higher American Society of Anesthesiologists (ASA) grades (p’s<0.001). Delirium was independently associated with a two-fold increased risk of inpatient mortality (adjusted odds ratio (aOR) 2.26, 95% confidence interval (CI) 1.79-2.84) and one-year mortality (aOR 2.05, 95%CI 1.83-2.29) and a lower likelihood of returning home within 30 days (aOR 0.27, 95%CI 0.24-0.30).

**Conclusion:**

Delirium affects around 20% of patients presenting with a hip fracture and is associated with important adverse outcomes. Integrating delirium assessment into the initial clinical assessment of hip fracture patients is feasible at national scale and should be considered as part of care for all hip fracture patients.

## Introduction

Delirium is a severe neuropsychiatric syndrome, common in older adults with hip fracture.^1, 2^ It is associated with adverse outcomes, including reduced mobility, prolonged hospital stay, higher mortality, higher post-discharge care requirements, higher risk of periprosthetic fracture, and causes distress and long-term psychological harms in patients and caregivers.^3–5^

Most studies of delirium in hip fracture have focused on postoperative delirium (POD),^6–9^ with fewer examining preoperative delirium. Studies involving delirium assessments performed prospectively by research teams reported preoperative prevalences between 16.4% and 57.6%.^10–17^ Two larger studies using chart review for retrospective delirium ascertainment reported preoperative prevalences of 7.8%^18^ and 12.7%.^6^ Several of these studies reported that preoperative delirium is associated with poor short-term outcomes, including higher inpatient mortality and longer hospital stay. Few studies have looked at outcomes after the acute inpatient episode, such as return home, identified by older adults as a top priority post-surgery.^19^

The authors previously studied delirium prevalence and outcomes in consecutive hip fracture admissions to a single trauma centre, with assessments performed in the Emergency Department (ED) or on ward admission.^3^ Delirium prevalence was 14.1% among patients admitted from home, 68.8% from care homes, and 26.5% overall. Delirium was associated with higher mortality, need for post-acute inpatient rehabilitation, and 180-day hospital readmission, after adjusting for important confounders.^3^ However, single-centre good practice is not always replicated. National audit data facilitates study of the feasibility and outcomes of preoperative delirium assessment at scale, across centres with varying caseloads, staffing and geriatrics specialists.

National hip fracture guidelines recommend delirium assessment,^20–22^ with early detection considered a requirement for good care.^23^ Some national hip fracture registries collect routine delirium assessment data and several recommend using the 4AT (www.the4AT.com), a simple 2-minute tool.^24–27^ However, there is variability in the timing of assessment. The Scottish Hip Fracture Audit (SHFA) is currently the only registry with published routine delirium assessment data from the time of presentation,^25^ while others, including the National Hip Fracture Database (NHFD), Irish Hip Fracture Database (IHFD) and Spanish Hip Fracture Registry (RNFC) recently began collection of preoperative delirium data. Delirium detection on presentation aligns with emergency care guidelines, which recommend assessment for all older patients in the ED or on admission.^23, 28^ Early identification of risk factors for adverse outcomes in older hip fracture patients is a top priority and can inform management.

This study examined the prevalence and outcomes of delirium in a large national sample of patients at the time of their presentation with a hip fracture. The main hypothesis is that delirium detected as part of routine care is independently associated with mortality and return to original residence within 30 days.

## Methods

### Study Design

This retrospective observational study used clinical registry data linked to national hospital admissions and mortality records. Approval was obtained from NHS National Services Scotland (ID DP22230478).

### Data Sources and Participants

The SHFA (www.shfa.scot.nhs.uk/) is a national registry covering >99% of people aged ≥50 hospitalized with acute hip fractures in Scotland. Data was obtained for all patients between 01/07/2019 and 31/12/2021. Hip fracture is defined in the SHFA as an intracapsular or extracapsular fracture of the proximal femur, up to and including subtrochanteric (the region extending 5cm below the lesser trochanter). Fractures around existing implants and isolated pubic ramus, acetabulum or greater trochanter fractures were excluded. Variables included: demographics; pre-fracture residence; hospital site; admission date; American Society of Anesthesiologists (ASA) grade; inpatient care factors including adherence to 12 care standards;^21^ length of stay; inpatient mortality; discharge destination and return to original residence within 30 days. People admitted from higher care settings including hospital, inpatient transfers and rehabilitation facilities but not care homes were excluded from analysis of return to original residence as it was not possible to accurately determine their original place of residence (own home, care home) using the available data or whether they had an increase in care needs post-hip fracture. The number of patients excluded on this basis was 700 (3.9%).

Individual-level audit data were linked by a unique identifier to national mortality records and Scottish Morbidity Records (SMR01), a hospital episode-based patient record, and by residence postcode to the Scottish Index of Multiple Deprivation (SIMD), a small-area based measure of relative social deprivation.^29^ All patients had at least one year follow-up data from admission. Processes for data extraction, validation and linkage are in Supplementary Figure 1.

### Delirium Assessment

Delirium assessment was performed prospectively by clinicians using the 4AT (www.the4AT.com), a brief (<2minute) tool that does not require specialist training.^30^ The 4AT is extensively validated, with a meta-analysis of 17 studies (3,702 observations) demonstrating a pooled sensitivity of 88% and pooled specificity of 88%.^31^ The score was categorized as per instructions: 4AT 0 (no delirium), 4AT 1-3 (suggestive of cognitive impairment, no delirium) and 4AT ≥4 (possible delirium, with or without cognitive impairment). The 4AT has been included in routine SHFA data collection since 01/07/2019. For audit purposes, assessment is performed at two timepoints: in the ED, and on the admitting orthopaedic ward within 24 hours.^21^ For each patient, the 4AT score from the ED was used; if none recorded, the first 4AT from the admitting orthopaedic ward. Patients with neither score were classified as no assessment.

### Outcomes

Outcomes were mortality and return to original residence within 30 days following admission. We determined the odds of mortality at two timepoints: inpatient (within 30 days) and within one year.

### Statistical Analysis

Continuous data were compared by ANOVA or Kruskal-Wallis tests, categorical variables by chi-squared tests, and differences between 4AT groups by paired comparisons tests with Bonferroni adjustment for multiple testing. An unadjusted Kaplan-Meier survival analysis was performed to estimate survival up to one year, stratified by delirium status. Multilevel mixed-effects logistic regression was used with 4AT 0 as the reference group to assess associations between 4AT score and mortality or return to residence, adjusting for age, sex, pre-fracture residence (home, care home, higher care setting), ASA grade, and SIMD quintile, with hospital site entered as a random intercept to account for clustering. Patients with no 4AT assessment were excluded from multivariable analysis. People without an ASA grade and/or linkable SIMD quintile were excluded from multivariable analyses as numbers were small. Covariates were specified *a priori* based on relevance and availability in routine SHFA data, and assumptions of logistic regression were tested and satisfied.

Statistical analyses were performed using R version 4.3.1 and packages.

### Supplementary Analyses

Univariable and multivariable analyses were performed to examine associations with return to original residence within 30 days, excluding patients who died within this period.

Descriptive analyses examined delirium prevalence and outcomes in patients managed non-operatively.

## Results

A total of 18,040 patients (mean age 80 years; 70% women) presented with a hip fracture. Of these, 16,476 (91%) had at least one 4AT assessment from the ED or orthopaedic ward within 24 hours, and 3,386 (21%) had scores suggestive of delirium. The first score was completed in the ED for 11,767 (71%) patients. ASA grades were missing for 314 patients (2%) and SIMD quintiles for 482 (3%). There were no other missing data.

Baseline characteristics are shown in Table 1, stratified by 4AT group. All characteristics differed significantly between groups. Compared to those without delirium (4AT 0), patients with delirium (4AT≥4) were older, more likely to be women, residing in care homes or higher care settings and had higher ASA grades (all p<0.001). Patients with no 4AT assessment were more likely to be men, transferred from higher care settings, living in more socioeconomically deprived areas, and had higher ASA grades (all p<0.001).

There was variation in 4AT completion and positive score rates across hospitals. For example, 21% of patients with no recorded assessment were from one centre that admitted 10% of the study population (Supplementary Table 1).

### Outcomes

Outcomes by 4AT group for patients with a recorded 4AT score are shown in Table 2. There were 700 patients from higher care settings excluded from analysis of return to residence. In unadjusted analyses, patients with 4AT≥4 had higher mortality risks as an inpatient and at one year and were less likely to return to their original residence within 30 days, compared to 4AT 0 (all p<0.001). Almost half (49%) of patients with 4AT≥4 died within a year, compared to 18% of 4AT 0 patients. Patients with no assessment had similar mortality rates as those with 4AT 1-3 (suggestive of cognitive impairment, but not delirium) (Supplementary Table 2).

**Figure 1:**
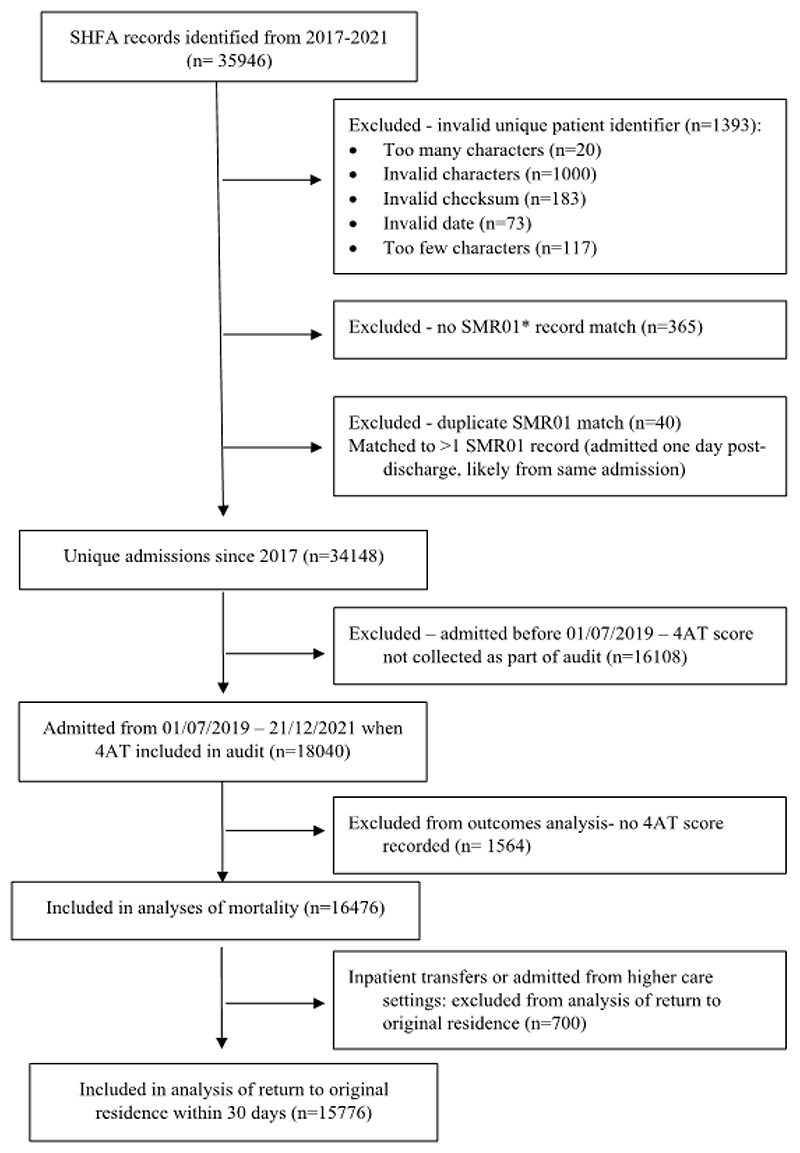
Unadjusted Kaplan-Meier survival curve showing survival up to one year, stratified by delirium status.

The Kaplan-Meier survival curves demonstrated lower survival rates among patients with delirium compared to those without over the one-year follow-up period, with the most marked difference in the initial period following hip fracture (Figure 1).

In multivariable analyses, 4AT≥4 was associated with two-fold increased odds of inpatient mortality (adjusted Odds Ratio (aOR) 2.26, 95% CI 1.79-2.84) and one-year mortality (aOR 2.05, 95% CI 1.83-2.29), and lower odds of returning to original residence within 30 days (aOR 0.27, 95% CI 0.24-0.30), compared to 4AT 0 ((Table 3).

Patients with 4AT 1-3 had lower mortality risks than those with 4AT≥4, but higher than 4AT 0 (inpatient: aOR 1.85, 95% CI 1.50-2.28; one year: aOR 1.69, 95% CI 1.54-1.86), and were less likely to return to their original residence (aOR 0.36, 95% CI 0.33-0.39).

### Supplementary Analyses

Sensitivity analysis including only people surviving to 30 days was consistent with the primary analysis (Supplementary Table 3).

There were 463 patients (3%) managed non-operatively. They had a higher delirium prevalence (28%) and higher mortality (Supplementary Tables 4 and 5).

## Discussion

Delirium was detected on presentation in approximately one in five hip fracture patients using the 4AT. In multivariable analyses, 4AT ≥4, suggestive of delirium, was associated with two-fold higher odds of inpatient mortality and one-year mortality and a lower likelihood of returning home within 30 days.

Delirium prevalence in this study is broadly aligned with prior smaller studies where delirium was ascertained by research teams.^10–16^ It was higher than the 7.8% and 12.7% reported by studies using retrospective chart review, likely reflecting methodologic differences and population factors.^6, 18^ Overall, present findings support the need to detect delirium early in the presentation with a hip fracture and we demonstrate this is feasible at national scale using a well-validated tool such as the 4AT.

Many hip fracture guidelines recommend postoperative delirium assessment. Existing studies suggest POD rates may be around one-third higher than preoperative rates. Studies using registry data from the NHFD in England, Wales and Northern Ireland reported POD prevalences of 25%, 26% and 29% using the 4AT,^32–34^ while a US National Surgical Quality Improvement Program (NSQIP) study reported a 27% prevalence using retrospective chart review.^8^ These studies reported worse outcomes including higher 30-day and one-year mortality rates in patients with postoperative delirium. The few existing research studies that assessed delirium pre- and postoperatively in the same cohort found the vast majority of patients with preoperative delirium also have POD, making preoperative delirium a very strong risk factor for POD.^10, 12, 15, 16^ These findings suggest postoperative assessment alone is insufficient: delirium should also be assessed preoperatively. Pre- and postoperative delirium are likely not the same entity,^10, 11^ warranting tailored approaches to management.

Early delirium detection is important for many reasons, including identifying and treating causes (some reversible, *e.g*. opioid administration), reducing risk of complications such as dehydration, malnutrition and infection, informing prognostication, and enhancing communication with patients and carers.^1^ A delirium diagnosis should trigger assessment of exacerbating factors and identification and management of symptoms, including distress.^5^ Early delirium detection and management may reduce severity and duration of the delirium episode^35, 36^ and length of stay and associated inpatient costs.^37, 38^

Observed associations of preoperative delirium with adverse outcomes is consistent with previous smaller research studies.^10, 11^ Associations were significant after adjusting for age, sex, pre-fracture residence and ASA grade, non-modifiable risk factors available at presentation.^39–42^ Further to previous studies, we also demonstrate these associations in patients managed non-operatively. While existing strategies to mitigate delirium have focused on intraoperative factors and preventing postoperative complications, this study highlights the importance of considering preoperative factors. Some are modifiable, such as rehydration, careful opioid use, and early peripheral nerve block, which may reduce pain and opioid use and is feasible in some prehospital settings.^43–45^

Over 90% of patients had a recorded 4AT, exemplifying successful implementation of national guidelines and standardised delirium assessment at scale. However, we observed hospital-level variation in delirium assessment and positive score rates. National registries like the SHFA and NHFD and quality improvement programs such as NSQIP can help to identify hospital-level variation and work towards greater standardisation of care, which is associated with better outcomes.^46, 47^

Delirium assessment on presentation should be routine for all older hip fracture patients, as in UK practice.^21^ We recommend using a validated assessment tool that is quick and effectively implemented by non-specialist clinicians, such as the 4AT. Early delirium detection facilitates proactive onward care planning and identification of modifiable clinical risk factors to ameliorate delirium and adverse outcomes. This approach may benefit other high-risk emergency surgical populations.^48^

Healthcare systems providing hip fracture care should analyse and report their routine delirium assessment data, examining completion and positive score rates. This is essential for effective, equitable delirium detection. Where assessment is not performed, the reason should be clearly documented.

Future studies should seek to identify inpatient care and perioperative factors that may modify or mediate associations between preoperative delirium and adverse outcomes, for example analgesic administration, time to surgery, and early mobilisation. This can inform care pathways and targeted interventions to improve outcomes. This study also raises important questions surrounding the decision to operate in some patients: of the 93% of patients managed non-operatively who were ASA grade 5 or “not ever fit for theatre”, 55% survived to discharge and 31% to a year. Knowledge of the increased relative risk associated with high ASA grade should be balanced by recognition that absolute risk of peri-operative mortality remains around 1% even in this high-risk group.^49^ Our study’s demonstration of the increased relative risk associated with preoperative delirium should obviously not prevent patients with delirium receiving surgery, a vital step in relieving their pain and distress.

### Strengths and Limitations

Key strengths include the use of national registry data in a universal healthcare setting, with near-complete (>90%) delirium assessment as part of routine care using a well-validated tool with high sensitivity and specificity.^31^ 4AT positive rates were consistent with preoperative delirium rates in this population estimated from prior research studies.

We note several limitations. This retrospective study used data routinely collected by the audit, and we were unable to specifically measure or control for confounders present on admission including dementia, fracture type, specific comorbidities or frailty. These unmeasured factors may account for some of the observed associations between delirium, mortality, and return home. Linkage to other routine health data (*e.g*. primary care records, disease registries) could help identify these and other important confounders, although they are not always recorded in routine data or available at time of presentation. Furthermore, the primary purpose of the multivariable model was not to draw causal inference but to understand the prognostic effect of delirium, adjusting for other routinely collected prognostic factors such as ASA grade. The SHFA is now collecting routine frailty data, which will allow future direct control for frailty. SIMD was determined using postcode at time of admission, which may not appropriately reflect SIMD for care home patients (18% of population). However, most people (76%) were admitted from their own homes, and pre-admission residence was used to determine SIMD for transfers from higher care settings (6%). Data on return to original residence is only collected at 30 days; other patients will ultimately return home following a longer admission or rehabilitation. Planned linkage to residence data will facilitate future study of this. Patients admitted from higher care settings may not have an ED 4AT assessment; however, these patients should still have an assessment recorded on the orthopaedic ward within 24 hours.^21^

## Conclusion

Delirium was detected in one in five hip fracture patients using routine 4AT assessment. Delirium was associated with higher mortality and lower likelihood of returning home. Future studies should focus on identifying modifiable pre-admission and inpatient care factors to ameliorate delirium and its harmful outcomes in this high-risk, high-volume emergency surgical population.

## Supplementary Material

Figure 1 & Tables i-v

## Figures and Tables

**Table 1:** Baseline characteristics of study cohort, by 4AT group.

	Whole cohort (n=18040)	No delirium (4AT 0) (n=8995)	Probable cognitive impairment (4AT 1-3) (n=4095)	Possible delirium (4AT ≥4) (n=3386)	No assessment (n=1564)	p-value
**Age** (mean (SD))	80 (10)	78 (10)	83 (9)	85 (8)	78 (12)	F-value = 562.1, df=3, p<0.001 (ANOVA)
**Sex** (female)	12594 (70)	6349 (71)	2796 (68)	2453 (72)	996 (64)	χ^2^=46.1, df=3, p<0.001
**Pre-fracture residence**						
Own home	13790 (76)	8640 (96)	2954 (72)	1263 (37)	933 (60)	χ^2^=6224.4, df=6, p<0.001
Care home	3157 (18)	200 (2)	912 (22)	1807 (53)	238 (15)	
Higher care setting^†^	1093 (6)	155 (2)	229 (6)	316 (9)	393 (25)	
ASA grade*						
1 (healthy)	314 (2)	253 (3)	27 (1)	14 (0)	20 (1)	χ^2^=1811.3, df=12, p<0.001
2	3945 (22)	2896 (33)	536 (13)	255 (8)	258 (17)	
3	10303 (58)	4805 (54)	2645 (66)	2032 (61)	821 (55)	
4	2763 (16)	783 (9)	755 (19)	900 (27)	325 (22)	
5 (moribund) or not ever fit for theatre^‡^ *314 missing	401 (2)	133 (2)	75 (2)	115 (4)	78 (5)	
SIMD Quintile**						
1 (most deprived)	3615 (21)	1663 (19)	924 (23)	672 (20)	356 (23)	χ^2^=60.355, df=12, p<0.001
2	3608 (21)	1794 (21)	875 (22)	615 (19)	324 (21)	
3	3716 (21)	1877 (22)	808 (20)	700 (21)	331 (22)	
4	3315 (19)	1703 (20)	689 (17)	666 (20)	257 (17)	
5 (least) **482 missing	3304 (19)	1676 (19)	705 (18)	654 (20)	269 (18)	
**Acute length of stay in days**						
median (IQR)	9 (9)	9 (8)	1 0 (9)	9 (8)	9 (9)	χ^2^=63.1, df=3, p<0.001 (Kruskal-Wallis)
**Discharge destination**						
Home	6846 (38)	5171 (58)	876 (21)	285 (8)	514 (33)	χ^2^=5558.5, df=9, p<0.001
Care home	2712 (15)	173 (2)	761 (19)	1542 (46)	236 (15)	
Higher care setting^†^	7549 (42)	249 (38)	2196 (54)	1263 (37)	688 (44)	
Died in hospital	933 (5)	249 (3)	262 (6)	296 (9)	126 (8)	

All in n (%) unless otherwise specified. p-values are for differences between groups assessed using Chi-squared tests unless otherwise specified, with Bonferroni correction for multiple testing. Abbreviations: ASA: American Society of Anesthesiologists; SIMD: Scottish Index of Multiple Deprivation, ANOVA: analysis of variance, IQR: interquartile range.

† Higher care settings include inpatient transfers, other acute hospital or rehabilitation settings.

‡ Includes both ASA grade 5 and additional SHFA category “not ever fit for theatre”, which may include some delayed presentations and patients not suitable for surgery

**Table 2:** Outcomes of study cohort, by 4AT group.

	All cause in-hospital mortality (within 30 days) N (%)	p-value	All cause mortality at one year N (%)	p-value	Return to original place of residence within 30 days N (%)	p-value
**Whole cohort** (n=16476)	845 (5)		5399 (33)		10333 (63) *	
**Delirium assessment**						
No delirium (4AT 0)	227 (3)	Ref	1639 (18)	Ref	6195 (70)	Ref
Probable cognitive impairment (4AT 1-3)	236 (6)	<0.001	1534 (38)	<0.001	1797 (47)	<0.001
Possible delirium (4AT ≥4)	272 (8)	<0.001	1661 (49)	<0.001	1637 (53)	<0.001

* 700 patients were admitted from higher care settings, including inpatient transfers, and were excluded from analysis of return to original residence within 30 days. p-values are chi-squared tests comparing each 4AT group with 4AT 0, with Bonferroni correction for multiple testing

**Table 3:** Results of mixed effects logistic regression models: unadjusted, and adjusted for age, sex, pre-fracture residence (home, care home, higher care setting), SIMD quintile and ASA grade, with hospital site entered as a random intercept to account for clustering.

	All cause in-hospital mortality (within 30 days)		All cause mortality at one year OR (95% CI)		Return to original place of residence within 30 days OR (95% CI)	
	Unadjusted OR (95% CI)	Adjusted OR (95% CI)	Unadjusted OR (95% CI)	Adjusted OR (95% CI)	Unadjusted OR (95% CI)	Adjusted OR (95% CI)
**4AT score**						
0	REF	REF	REF	REF	REF	REF
1-3	2.36 (1.96-2.85)	1.85 (1.50-2.28)	2.69 (2.47-2.92)	1.69 (1.54-1.86)	0.37 (0.34-0.40)	0.36 (0.33-0.39)
4+	3.37 (2.82-4.04)	2.26 (1.79-2.84)	4.32 (3.97-4.71)	2.05 (1.83-2.29)	0.49 (0.45-0.53)	0.27 (0.24-0.30)
**Age** (per year)	1.04 (1.03-1.05)	1.04 (1.03-1.05)	1.06 (1.05-1.06)	1.04 (1.04-1.05)	0.95 (0.95-0.95)	0.95 (0.94-0.95)
**Sex**						
Male	REF	REF	REF	REF	REF	REF
Female	0.54 (0.47-0.62)	0.52 (0.44-0.61)	0.59 (0.55-0.63)	0.50 (0.46-0.55)	1.31 (1.22-1.40)	1.42 (1.31-1.54)
**Pre-fracture residence**						
Own home	REF	REF	REF	REF	REF	REF
Care home	1.60 (1.35-1.89)	0.69 (0.56-0.86)	3.22 (2.97-3.48)	1.52 (1.36-1.69)	2.06 (1.89-2.25)	8.40 (7.42-9.50)
Higher care setting	2.24 (1.78-2.81)	0.89 (0.64-1.24)	3.34 (2.94-3.78)	1.83 (1.54-2.18)	-*	-*
**ASA grade**						
1 (healthy)	0.29 (0.09-0.92)	0.58 (0.18-1.82)	0.19 (0.13-0.29)	0.40 (0.26-0.62)	8.13 (5.43-12.18)	5.15 (3.31-8.00)
2	0.37 (0.27-0.50)	0.55 (0.40-0.76)	0.28 (0.25-0.31)	0.41 (0.37-0.47)	2.81 (2.58-3.07)	2.48 (2.24-2.74)
3	REF	REF	REF	REF	REF	REF
4	3.32 (2.81-3.92)	2.78 (2.30-3.36)	2.71 (2.49-2.95)	2.20 (1.99-2.43)	0.66 (0.60-0.72)	0.62 (0.56-0.69)
5 (moribund) or not ever fit for theatre	23.15 (18.46-29.03)	22.52 (17.23 - 29.43)	5.69 (4.57-7.09)	5.71 (4.39-7.43)	0.29 (0.23-0.37)	0.22 (0.17-0.30)
**SIMD Quintile**						
1 (most deprived)	REF	REF	REF	REF	REF	REF
2	1.08 (0.88-1.33)	1.11 (0.87-1.42)	1.04 (0.92-1.17)	1.02 (0.91-1.14)	0.89 (0.81-0.99)	0.96 (0.85-1.08)
3	0.89 (0.71-1.10)	0.83 (0.64-1.08)	0.88 (0.78-0.99)	0.87 (0.78-0.98)	0.94 (0.85-1.04)	0.99 (0.88-1.11)
4	0.86 (0.68-1.08)	0.90 (0.69-1.17)	0.91 (0.80-1.03)	0.89 (0.79-1.00)	1.00 (0.91-1.11)	1.00 (0.89-1.13)
5 (least deprived)	0.91 (0.73-1.14)	0.96 (0.74-1.25)	0.89 (0.78-1.01)	0.89 (0.79-1.00)	1.07 (0.97-1.18)	1.09 (0.96-1.23)

Abbreviations: OR: odds ratio, CI: confidence interval, ASA: American Society of Anesthesiologists, SIMD: Scottish Index of Multiple Deprivation.

* Inpatient transfers and patients admitted from higher care settings including hospitals and rehabilitation facilities excluded from return to original residence within 30 days. NB covariate adjustment set is for effect of 4AT score on outcomes and coefficients for other variables cannot be interpreted from these models.
