# Supplementary material for: Delirium on presentation with a hip fracture is associated with adverse outcomes: a multicentre observational study of 18,040 patients using national clinical registry data
Source: Bone Joint J. Author manuscript; Available in PMC 2025 May 14. (PMC7617671; doi:10.1302/0301-620X.107B4.BJJ-2024-1164.R1)
Supplement: Figure 1 & Tables i-v [file EMS204316-supplement-Figure_1___Tables_i_v.pdf]

The Bone & Joint Journal

Supplementary Material

10.1302/0301-620X.107B3.BJJ-2024-1164.R1

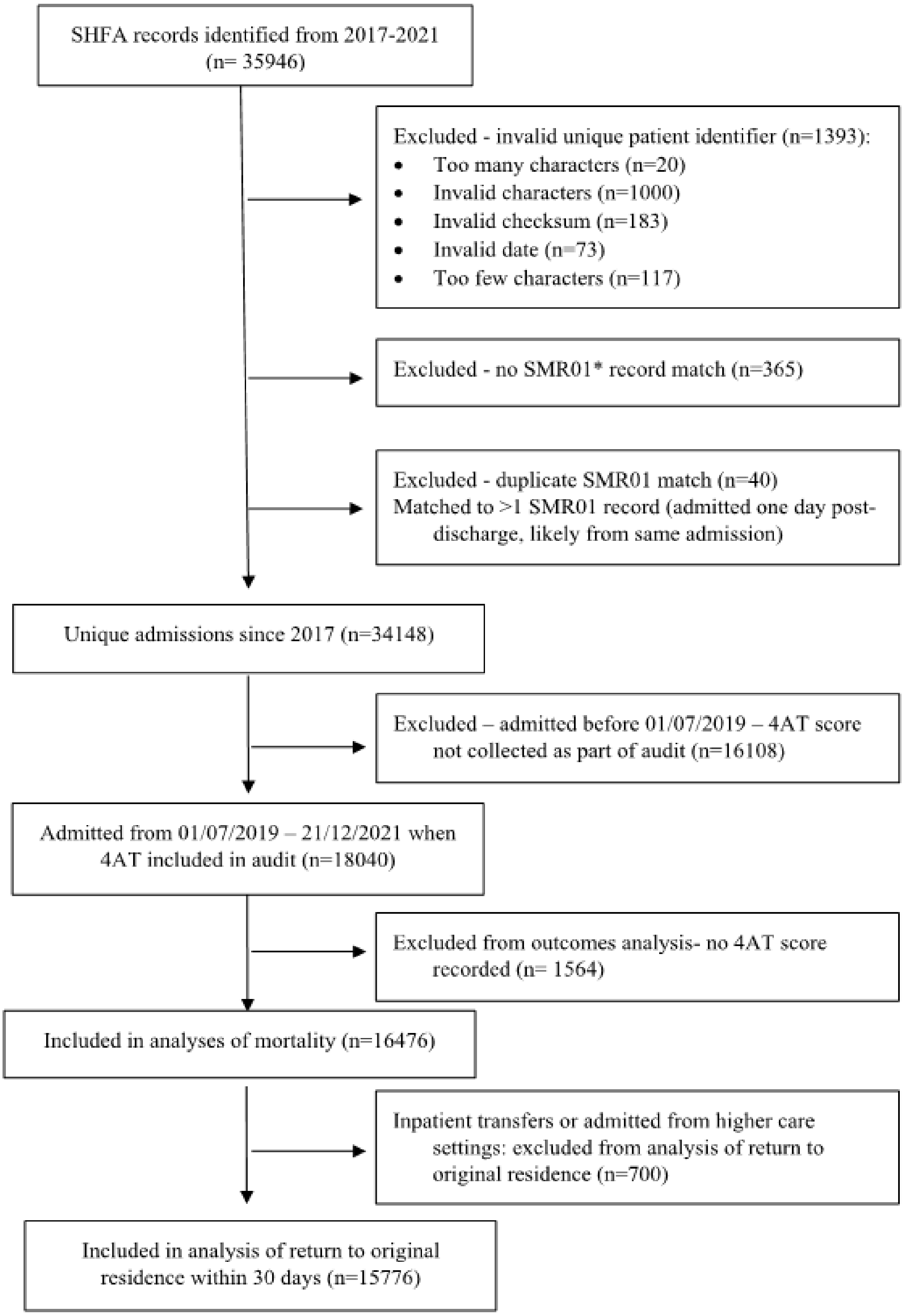

Fig. a. National registry data extraction, cleaning, and linkage process. *SMR01 is an episode-based patient record of all inpatient and day cases discharged from non-obstetric and non-psychiatric specialties in Scotland. SHFA, Scottish Hip Fracture Audit; SMR, Scottish Morbidity Records.

Table i. Variation in 4AT scores by hospital site. Each hospital site was assigned an anonymized code (1 to 20). All data shown as n (%).

Hospital site	Whole cohort (n = 18040)	No delirium (4AT 0) (n = 8995)	Probable cognitive impairment (4AT 1 to 3) (n = 4095)	Possible delirium (4AT ≥ 4) (n = 3386)	No assessment (n = 1564)	p-value (Chi-square test)
1	883 (5)	459 (5)	229 (6)	165 (5)	30 (2)	< 0.001
2	499 (3)	275 (3)	112 (3)	90 (3)	22 (1)	
3	427 (2)	214 (2)	99 (2)	82 (2)	32 (2)	
4	387 (2)	184 (2)	84 (2)	92 (3)	27 (2)	
5	1083 (6)	545 (6)	265 (7)	225 (7)	48 (3)	
6	1103 (6)	518 (6)	287 (7)	239 (7)	59 (4)	
7	1277 (7)	599 (7)	312 (8)	242 (7)	124 (8)	
8	1806 (10)	898 (10)	409 (10)	324 (10)	175 (11)	
9	820 (5)	433 (5)	150 (4)	158 (5)	79 (5)	
10	257 (1)	135 (2)	57 (1)	42 (1)	23 (2)	
11	1619 (9)	777 (9)	422 (10)	315 (9)	105 (7)	
12	1405 (8)	721 (8)	261 (6)	305 (9)	118 (8)	
13	363 (2)	224 (3)	70 (2)	60 (2)	9 (1)	
14	2575 (14)	1360 (15)	520 (13)	472 (14)	223 (14)	
15	1719 (10)	745 (8)	326 (8)	319 (9)	329 (21)	
16	2 (0)	0 (0)	0 (0)	0 (0)	2 (0)	
17	1022 (6)	499 (6)	326 (8)	119 (4)	78 (5)	
18	130 (1)	64 (1)	18 (0)	29 (1)	19 (1)	
19	642 (4)	336 (4)	143 (4)	102 (3)	61 (4)	
20	21 (0)	9 (0)	5 (0)	6 (0)	1 (0)	

Table ii. Outcomes of the 1564 patients with no recorded 4AT delirium assessment.

All-cause in-hospital mortality (within 30 days), n (%)	All-cause mortality at one year, n (%)	Return to original place of residence within 30 days, n (%)
115 (7)	565 (36)	705 (60)

Table iii. Results of mixed effects logistic regression model, including only people who survived to 30 days: unadjusted and adjusted for age, sex, pre-fracture residence (home or care home), SIMD quintile, and ASA grade, with hospital site entered as a random intercept to account for clustering.

	**Return to original place of residence within 30 days**	
	**Unadjusted OR (95% CI)**	**Adjusted OR (95% CI)**
**4AT score**		
0	REF	REF
1 to 3	0.39 (0.36 to 0.43)	0.36 (0.33 to 0.40)
4+	0.62 (0.57 to 0.68)	0.28 (0.24 to 0.32)
**Age (per year)**	0.95 (0.95 to 0.96)	0.95 (0.94 to 0.95)
**Sex**		
Male	REF	REF
Female	1.21 (1.13 to 1.31)	1.31 (1.20 to 1.43)
**Pre-fracture residence**		
Home	REF	REF
Care home	3.98 (3.54 to 4.47)	15.60 (13.39 to 18.17)
**ASA grade**		
1 (healthy)	10.07 (6.24 to 16.25)	6.53 (3.92 to 10.91)
2	2.70 (2.47 to 2.96)	2.52 (2.27 to 2.80)
3	REF	REF
4	0.81 (0.74 to 0.89)	0.69 (0.61 to 0.78)
5 (moribund) or not ever fit for theatre	0.95 (0.69 to 1.30)	0.70 (0.47 to 1.04)
**SIMD quintile**		
1 (most deprived)	REF	REF
2	0.88 (0.79 to 0.98)	0.95 (0.84 to 1.08)
3	0.92 (0.83 to 1.02)	0.95 (0.84 to 1.08)
4	0.98 (0.88 to 1.10)	0.97 (0.85 to 1.11)
5 (least deprived)	1.07 (0.96 to 1.20)	1.08 (0.95 to 1.24)

Inpatient transfers and patients admitted from higher care settings including hospitals and rehabilitation facilities were excluded from return to original residence within 30 days.

ASA, American Society of Anesthesiologists; SIMD, Scottish Index of Multiple Deprivation.

Table iv. Baseline characteristics of hip fracture patients managed nonoperatively, by 4AT group (n = 463). All data are shown as n (%) unless otherwise specified.

Characteristic	Whole cohort (n = 463)	No delirium (4AT 0) (n = 154)	Probable cognitive impairment (4AT 1 to 3) (n = 84)	Possible delirium (4AT ≥ 4) (n = 128)	No assessment (n = 97)
Mean age, yrs (SD)	80 (10)	77 (11)	84 (9)	84 (9)	77 (11)
Sex (female)	274 (59)	95 (62)	49 (58)	82 (64)	48 (50)
**Pre-fracture residence**					
Home	304 (66)	136 (88)	54 (64)	57 (45)	57 (59)
Care home	102 (22)	7 (5)	23 (27)	60 (47)	12 (12)
Higher care setting*	57 (12)	11 (7)	7 (8)	11 (9)	28 (29)
**ASA grade†**					
1 (healthy)	1 (0)	1 (1)	0 (0)	0 (0)	0 (0)
2	4 (1)	4 (3)	0 (0)	0 (0)	0 (0)
3	8 (2)	3 (2)	2 (3)	0 (0	3 (4)
4	18 (4)	5 (4)	1 (1)	6 (5)	6 (7)
5 (moribund) or not ever fit for theatre‡	385 (93)	126 (91)	73 (96)	110 (95)	76 (89)
**SIMD quintile§**					
1 (most deprived)	110 (25)	43 (30)	19 (23)	27 (21)	21 (22)
2	93 (21)	23 (16)	19 (23)	33 (26)	18 (19)
3	90 (20)	30 (21)	16 (19)	26 (21)	18 (19)
4	79 (18)	26 (18)	14 (17)	16 (13)	23 (25)
5 (least deprived)	76 (17)	23 (16)	15 (18)	24 (19)	14 (15)
Median acute length of stay, days (IQR)	5 (2-10)	6 (2-14)	4 (2-9)	4 (2-8)	5 (2-9)
**Discharge destination**					
Home	95 (21)	58 (38)	13 (16)	8 (6)	16 (17)
Care home	51 (11)	4 (3)	9 (11)	33 (26)	5 (5)
Higher care setting*	110 (24)	40 (26)	22 (26)	22 (17)	26 (27)
Died in hospital	207 (45)	52 (34)	40 (48)	65 (51)	50 (52)

*Higher care settings include inpatient transfers, other acute hospital or rehabilitation settings.

†47 missing.

‡Includes both ASA grade 5 and additional SHFA category “not ever fit for theatre”, which may include some delayed presentations and patients not suitable for surgery.

§15 missing.

ASA, American Society of Anesthesiologists; SIMD, Scottish Index of Multiple Deprivation.

Table v. Outcomes of hip fracture patients managed nonoperatively, by 4AT group.

	All-cause in-hospital mortality (within 30 days), n (%)	All-cause mortality at one year, n (%)	Return to original place of residence within 30 days, n (%)
Whole cohort (n = 463)	201 (43)	321 (69)	117 (29)
**Delirium assessment**			
No delirium (4AT 0) (n = 154)	48 (31)	81 (53)	51 (36)
Probable cognitive impairment (4AT 1 to 3) (n = 84)	40 (48)	65 (77)	17 (22)
Possible delirium (4AT ≥ 4) (n = 128)	64 (50)	105 (82)	28 (24)
